# Long-Term Immunocastration Protocols Successfully Reduce Testicles’ Size in Bísaro Pigs

**DOI:** 10.3390/ani11030632

**Published:** 2021-02-27

**Authors:** Gustavo Paixão, Sofia Botelho Fontela, Jorge Marques, Alexandra Esteves, Rui Charneca, Rita Payan-Carreira

**Affiliations:** 1 Animal and Veterinary Research Centre (CECAV), Universidade de Trás-os-Montes e Alto Douro, 5000-801 Vila Real, Portugal; sbotelho@utad.pt (S.B.F.); alexe@utad.pt (A.E.); 2 MED-Mediterranean Institute for Agriculture, Environment and Development & Department de Medicina Veterinária, Universidade de Évora, Pólo da Mitra, 7002-554 Évora, Portugal; jfcm.3mai@gmail.com (J.M.); rmcc@uevora.pt (R.C.)

**Keywords:** boar, improvac, scrotal dimensions, immunosuppression, pork

## Abstract

**Simple Summary:**

Surgical castration is an ongoing problem in pig production, especially in breeds reared until older ages, like the Bísaro pig. To avoid this technique, three immunocastration protocols with different numbers of injections and times of inoculation were tested. Scrotal measurements were used to attest their effectiveness. The best protocol was the one with three administrations starting at three months old. It was able to maintain the testis size below puberty size for 16 weeks following the last injection.

**Abstract:**

This study aimed to find a suitable immunocastration protocol for male Bísaro pigs (BP) due to the breed and production system particularities. Twenty-five male BP were treated with Improvac^®^ according to three protocols: using two (GrpE2 and L2) or three vaccinations (GrpL3) and starting at 9 (GrpE2) or 13 weeks old (GrpL2 and L3). Eleven animals were kept as intact males (GrpC). Scrotal measurements and the morphometry of the testes and epididymides collected at slaughter were used to survey the effectiveness of the immunocastration compared with the age-matched intact controls. Animals in groups E2 and L3 were kept until 57 weeks, after a second vaccination cycle at 49 and 53 weeks of age. Scrotal dimensions decreased to almost initial values in treated animals until 17 (GrpE2) and 21 weeks (GrpL2 and L3), thereafter increasing to post-pubertal values until around 29 or 37 weeks of age for groups E2 and L2, respectively, but only at 41 weeks in group L3. Between 41 and 49 weeks, scrotal dimensions were similar in treated and control animals, decreasing to the predicted pre-puberty size after the second cycle of vaccination. This study suggests the most suited protocol for males slaughtered at older ages includes three administrations of Improvac^®^ starting at 3 months of age, followed by a second vaccination cycle.

## 1. Introduction

Animal welfare, in particular surgical castration, has been a major concern for the European pork industry in the last decade. In 2010, the European Commission issued a declaration on alternatives to the surgical castration of pigs [1] and committed to stopping castration by 2018. Goals from this declaration are far from being met [2], and the EU has not yet banned pig castration. As this procedure is traditionally done without anesthesia or analgesia, it is undeniably associated with pain, stress, and the risk of infection, thus promoting the use of alternatives [3,4,5,6]. Consumers also share similar welfare concerns [7]. A promising alternative is immunocastration, which is known for suppressing gonadal activity and producing carcasses without boar taint [8,9]. This procedure is based on the administration of a gonadotropin-releasing hormone analogue (GnRH) conjugated to a protein [10], which drives the formation of antibodies against GnRH, thereby preventing luteinizing hormone (LH) and follicle-stimulating hormone (FSH) secretion [11]. Consequently, the secretion of testicular steroids, such as androstenone, one of the main compounds responsible for boar taint [12], is reduced. Another compound is skatole, produced in the hindgut by microbial activity from tryptophan and its metabolism modulated by gonadal steroids in the liver [13]. Besides this, immunocastrated animals will have reduced fat accumulation compared to surgically castrated ones. The differences in fat accumulation have been recently associated with FSH regulation of the genes balancing lipogenesis/lipolysis. In surgically castrated pigs, high FSH levels drive a reduction of lipolysis, favoring fat accumulation, while in immunocastrated pigs, the reduced FSH levels have the opposite effect [14]. This technique appears to be broadly accepted as a valid alternative for local breeds [15,16] preferred by consumers regarding their perceived health-related risks [17,18], with no significant consequences for the meat quality or cured products [16,19,20].

Even so, immunocastration still presents many uncertainties. In the case of Bísaro pigs (BP), like some other breeds produced in traditional extensive systems in the Mediterranean area, the animals are usually reared up to ages of more than a year old, often in mixed gender groups. Moreover, many systems are based on or include periods of extensive grazing [21]. These periods entail limited handling facilities and the risk of the unintentional mating of females with male cohorts or wild boars. These factors suggest that immunocastration should start as early as is required and be maintained for longer periods, in contrast with the manufacturer’s short-term protocol for industrial systems.

The reproductive capability of male pigs can be predicted by scrotal measurements [15,22,23]. Puberty is a period characterized by rapid reproductive development and may be perceived by the increase in testicular growth. On the other hand, the effectiveness of immunocastration can be predicted indirectly through testicular measurements [24,25,26] or the development of sexual accessory glands [27], as immunocastrated animals have less developed genital organs [28]. The morphometric analysis of scrotal gonads, considering pre- and post-pubertal values, can then be a useful tool when evaluating the effectiveness of immunocastration protocols.

The Bísaro pig is one of the most representative Portuguese autochthonous breeds [29,30] and is known for its robustness and reported meat quality [31]. The BP is sexually precocious, reaching puberty between 14 and 17 weeks old [32]. If intended for the industry of cured value-added products, it is mainly reared up to 13 months old. Yet today, a major limitation for the growth of this industry is that an increasing number of animals are slaughtered at younger ages, before or shortly after weaning (approx. 45 d) [21], as a result of various difficulties in complying with the castration restrictions introduced by the EU in 2008 and thus in rearing animals to older ages. This essay aims to define an appropriate immunocastration protocol to overcome this issue, allowing for an extended suppression period of testicular function with a minimal number of administrations. It will also allow the establishment of extended protocols suited for other autochthonous breeds reared in similar non-industrial systems.

## 2. Materials and Methods

### 2.1. Vaccination Protocols

**Figure 1:**
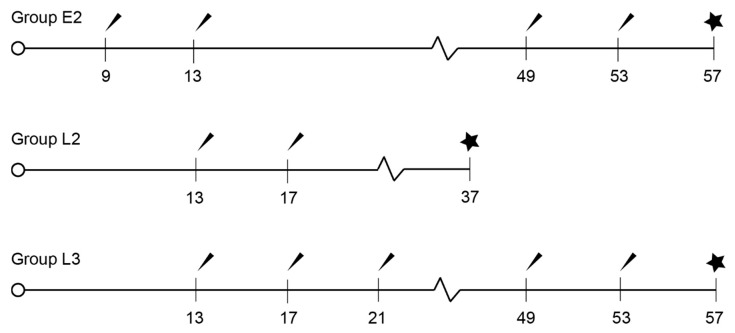
Scheme of the vaccination protocols (weeks). Arrow: injection; Star: slaughter.

Thirty-nine male Bísaro pigs were used in this study. Twenty-eight were randomly assigned to three treatment groups and one control group. Animals in the treatment group were vaccinated against GnRH with Improvac^®^ (Zoetis) with the standard dosing regimen (2 mL subcutaneous) using different protocols (Figure 1). In group E2, two animals died at the beginning of the trial and one was excluded after an accident. Priming started at 9 weeks in the early (E) group (GrpE2; *n* = 8) and at 13 weeks of age for the late (L) starters (GrpL3; *n* = 11 and GrpL2; *n* = 6). The booster was performed with one (GrpE2 and L2) or two injections (GrpL3) 4 weeks apart. Animals in groups E2 and L3 had a second vaccination cycle (2 shots) starting at 49 weeks of age (8 weeks before slaughter at 57 weeks of age). Animals in group L2 only had one vaccination cycle and were slaughtered at 37 weeks of age.

Litters were only selected from animals registered in the herdbook. The Bísaro pig is an indigenous breed with significant genetic variability and, consequently, phenotypic variability. Animals were from different litters and might have presented differences in the absolute values at the beginning of the experiment. Comparison of the relative change in scrotal dimensions of the three groups allows for the inference of the suppressive effects of the vaccine on the testicular function of the treated animals.

Eleven animals were kept as intact males throughout the whole experiment (GrpC; *n* = 11) to establish age-match scrotal dimensions. Animals in the control group were also maintained until 57 weeks old.

Animals were reared indoors in groups of 5–6 animals until 29 weeks old, and thereafter in outdoor pens with approximately 1000 m^2^ each, and fed ad libitum. All premises complied with the welfare requirements established in the Council Directive of 2008 [33].

The study was approved by the Animal Care and Ethical Committee of the University of Trás-os-Montes e Alto Douro (Protocol number 657-e-DZ-2018).

### 2.2. Measurements

Scrotal dimensions (total width, plus the height and width of the left and right scrotal sac) were measured every 4 weeks from the first injection (FI) until the 44th (GrpE2 and L3) and 24th (GrpL2) week post-administration. Due to operational issues at the experimental farm, measurements from the control group were not taken at 33 weeks old or from groups E2 and L3 at 37 weeks old. Measurements were performed based on the Davis and Hines method [34] using a vernier caliper. Briefly, with the animal standing, the total width was obtained at the largest horizontal transversal axis of the scrotum, the height of the scrotal sac was obtained at the higher vertical length of each scrotal sac, while the maximum horizontal diameter provided the scrotal sac width. A single operator conducted the procedures in order to minimize inter-operator biases. Animals were contained in a specially made handling crate, where they were simultaneously weighted.

Additionally, the testis and epididymis were collected after slaughter and the testicular and epididymal morphometric values were then taken (aggregated weight of testis and epididymis, testis weight, width and length; epididymal weight).

Absolute dimensions from in vivo and post-mortem measurements were compared to the expected pubertal dimensions of this specific breed [32].

### 2.3. Statistical Analysis

Gonadal measurements were grouped by animal. Thus, average values for both organs were calculated throughout the experiment [35]. The Shapiro–Wilk test was used to assess the normality of variables of interest.

Descriptive analysis was firstly performed to characterize the evolution of the scrotal size. The variation between each measurement in relation to the previous measurement was calculated to survey the immunosuppressive effects of the vaccination protocols. The variation was then compared between groups. Since the variables were not normally distributed, group differences were identified using a Wilcoxon rank test. A confidence level of 99% was used to minimize potential inaccuracies from in vivo measurements. The variation in normal scrotal growth (GrpC) was also compared with the treatment groups. A least squares model was used in the post-mortem analysis. Carcass weight and group were included as factors. Statistical analyses were performed using JMP 7 software (SAS Institute Inc., Cary, NC, USA).

## 3. Results

### 3.1. Absolute Dimensions of the Scrotal Sacs

**Figure 2:**
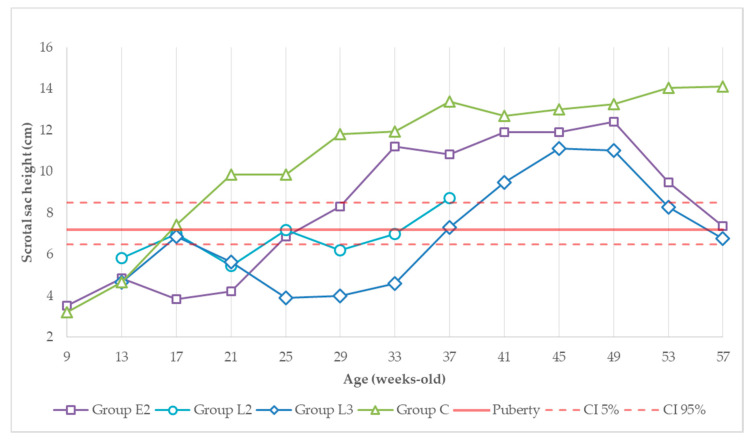
Evolution of scrotal sac height (cm) over experimental time. Red solid and dashed lines correspond to the predicted testicular length at puberty (95% confidence level) [32]. Dimensions from groups E2 and L3 in the 37th week and from the control group in the 33rd week were estimated.

**Figure 3:**
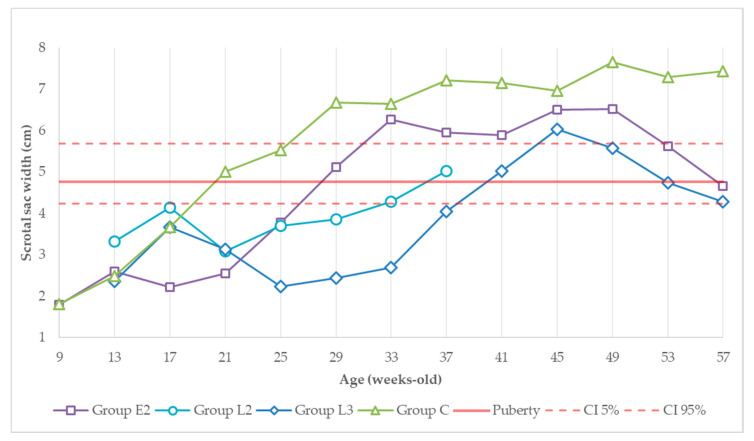
Evolution of scrotal sac width (cm) over experimental time. Red solid and dashed lines correspond to the predicted testicular width at puberty (95% confidence level) [32]. Dimensions from groups E2 and L3 in the 37th week and from the control group in the 33rd week are estimated.

Figure 2 and Figure 3 show the scrotal sac height and width evolution throughout the study. At the priming dose, values started at around 4 cm in height in nine-week-old animals and increased until they were 13 weeks old (GrpE2) or 17 weeks old (GrpL2, L3 and C). After the second administration, measurements decreased to values close to initial values in all treated animals until weeks 17 and 21 (GrpE2 vs. GrpL2 and L3). By then, the scrotal sac measurements increased and approximated the expected size of sexually mature animals. This process was slower in group L3, taking almost 16 weeks. The pre-established pubertal height and width were surpassed at 21 weeks of age in the control group, 29 weeks of age in group E2, and 37 weeks of age in group L2. Group L3 only overtook this value at 41 weeks old. Between weeks 41 and 49, scrotal dimensions in the treated animals became closer to those of the control group.

From the 49th week (first dose of the second vaccination cycle), scrotal dimensions from both treated groups decreased to values close to the predicted puberty mark. The total scrotal width followed the same pattern (data not shown). From the 53rd week (4 weeks after the first administration in the second vaccination cycle) until the last observation of the study, scrotal dimensions from the control group remained higher than in the re-treated groups E2 and L3.

### 3.2. Variation of Scrotal Dimensions

Variation of all scrotal dimensions did not differ significantly between the treatment groups after the first injection (*p* > 0.01) (4 weeks post FI) or from the onset of the treatment to the second administration of the vaccine, 8 weeks after FI (Table 1). At the 12th week post FI, however, the variation in all the measurements differed between the groups (*p* ≤ 0.01), as groups E2 and L2 were already reverting to the negative variation and group L3 was still on a negative tendency. While groups E2 and L2 already registered a considerable positive variation, group L3 still registered a negative variation. At 16 weeks after FI, there were still significant differences in the variation of the scrotal sac height and scrotal sac width (*p* ≤ 0.01), although less pronounced. From the 20th week from FI onwards, the predominantly positive variation in measurements became similar between the groups (*p* > 0.01).

Both group E2 and group L3 inverted the positive variation after the second vaccination cycle at 49 and 53 weeks of age, respectively. Responses did not differ significantly between the treated groups (Table 1).

### 3.3. Post-Mortem Morphometry

Table 2 summarizes the morphometric values taken after gonadal excision at slaughter. The average carcass weight at slaughter of the treated animals was 117.7 kg. Animals in group E2 were 8% and 5% heavier than animals in groups L2 and L3, respectively. Between the three treatment groups, no significant differences were found regarding testicular and epididymal post-mortem morphometry (*p* > 0.01), even though testis weight registered the most noticeable difference (*p* = 0.065). Aggregated weight, testis width, and epididymal weight differed slightly between the treated groups, but not statistically. When comparing with the control group, the differences became evident (*p* < 0.01). Testicular and epididymal dimensions were approximately 31% larger in intact animals than in treated animals. Gonads from entire males were 66% heavier than in treated animals. Animals in the control group also had heavier carcasses (12%).

## 4. Discussion

The present assay is the first, to our knowledge, to test immunocastration protocols in male Bísaro pigs and to demonstrate that this procedure could be an alternative method for the castration of these animals. All tested protocols achieved a reduction in the scrotal dimensions to pre-pubertal sizes, suggesting the success of the immunocastration process. A second cycle of Improvac^®^ was needed to sustain the inhibition of the reproductive function until slaughter, close to one year of age.

Earlier vaccination protocols have been studied and compared to the one recommended by the manufacturer to enhance the suppression of reproductive function. Some of the advantages are a distinct reduction in the reproductive organs’ size, a reduction in testicular activity and controlled boar taint [4,36]. Similarly to the results of Sladek et al. [36] and Brunius et al. [4], in this assay, the early vaccination performed in group E2, starting at 9 weeks of age, did not interfere with the vaccine’s efficacy. For sexually precocious breeds, like BP, this is of major importance.

For local breeds reared in non-industrial systems, keeping male pigs asexual during an extended rearing and fattening period is imperative. Therefore, the efficacy needs to be evaluated not only at earlier stages and at slaughter but also during the entire growing phase after puberty, which for BP starts as early as 14 weeks old [32]. Longer and post-pubertal protocols have been tested in autochthonous breeds, as they have different characteristics than industrial crosses. For example, Hernandez-Garcia et al. [37] successfully adapted a protocol for Iberian sows/gilts with three inoculations of Improvac^®^ at 10.5, 12, and 13.5 months, with slaughter at 16 months old. Gogic et al. [27] also succeeded in adapting the manufacturer’s protocol to a local breed, the Mangulica; the first vaccination was given to 275-day-old pigs and the second to approximately 320-day-old pigs (85 and 40 days prior to slaughter, respectively). In our study, the three-dose protocol kept gonads below pre-pubertal dimensions for a longer period of time compared to the two dose protocol, whether it started at around two or at around three months of age. Animals from group L2 were slaughtered earlier (37 weeks old); however, the post-mortem morphometric analysis showed that the testicles’ dimensions stayed below or near average pubertal values. However, for the treated groups kept in the farm, a second vaccination cycle was needed in order to have an immunocastration effect by slaughter time. This cycle started when the scrotal dimensions were above the predicted pubertal mark.

To evaluate the scrotal dimensions and their relationship with spermatogenesis and puberty, we used the predicted testicular dimensions at puberty and a control group of age-matched intact pigs. Paixão et al. [32] used post-mortem testicular measurements combined with histological evaluation to create a model that inferred testicular dimensions at the onset of puberty and sexual capability. Scrotal measurements include muscular fasciae, membranes, skin, and variable portions of epididymal tissue, besides the testicular dimensions. Therefore, surveilling the scrotum dimensions will allow to follow the changes in testicular size in response to immunocastration. Comparing the scrotal measurements with the predicted post-mortem testicular dimensions strengthens the information provided in this study.

It seems that male pigs recover gonadal dimensions faster than females. With a three-dose protocol at 19, 23, and 39 weeks old, Hernández-García et al. [37] were able to keep female gonads at pre-pubertal dimensions until slaughter at 68 weeks of age. This protocol kept the gilts immunocastrated for more than 6 months after the last injection. In our assay, the dimensions from male pigs fully inverted the negative variation 4 months after the last injection and recovered to similar gonadal dimensions of contemporaneous entire pigs in less than 20 weeks. It also confirms that immunocastration can be reversed, as theorized, once the antibody levels decrease to a minimum [38]. Whether or not sequela of spermatogenesis will remain is still uncertain. To optimize the vaccine’s potential, minimizing the costs and animal handling, other variations to the proposed protocol could also be tested. Given the data collected in this study, in particular, the marked negative scrotal dimension variation at 4 weeks post-FI in the second vaccination cycle, it appears that the second administration in the second vaccination cycle could be avoided. Perhaps it could be reserved for animals that do not respond adequately to the first injection. The 8 week period in which the scrotal dimensions of the treated animals were close to those of the non-treated animals could be considered a problem in alternative systems. To overcome this issue, we could have postponed the third injection or brought forward the second cycle to the 33rd or 37th week of age. Data analysis shows that an effective protocol for BP should start in 3-month-old male pigs with three injections of Improvac^®^, adding a second cycle of one or two administrations at 33/37 weeks of age. This protocol would better match the practices of the Bísaro production system, where producers seek to attain one-year-old animals at the time of slaughter, allowing them to maintain pre-pubertal testicular dimensions throughout most of the growing and fattening phase.

For locally bred pigs in non-industrial systems, sexual steroids are important not only at slaughter, but also for the suppression of sexual activity during all growing and fattening phases. Therefore, other alternatives to surgical castration, such as feeding strategies, breeding programs, or gene editing for leveling skatole and androstenone, seem insufficient for these particular systems. Nonetheless, steroid hormones and skatole leveling, along with histological analysis, should be performed to confirm the efficacy of the tested protocols. Carcass evaluation and meat quality comparison is also foreseen to validate the absence of boar taint at slaughter. These assays will allow for a better understanding of the protocols’ differences and how they should be implemented with local farmers.

## 5. Conclusions

All tested protocols successfully reduced testicles’ sizes compared to entire animals. For variable periods of time, dimensions were kept below those predicted in pubertal animals. In male Bísaro pigs, the immunocastration protocol with three inoculations of Improvac^®^ kept testicles below pubertal dimensions for a longer period of time, implying a more consistent and prolonged effect.

## Figures and Tables

**Table 1:** Mean variation of scrotal sac height and width over time (weeks after first injection).

Weeks after FI	Scrotal Sac Height				Scrotal Sac Width			
	Grp E2	Grp L2	Grp L3	*p*-Value	Grp E2	Grp L2	Grp L3	*p*-Value
4	0.38	0.20	0.48	0.020	0.47	0.26	0.61	0.216
8	−0.21	−0.22	−0.17	0.714	−0.14	−0.26	−0.11	0.053
12	0.08 ^b^	0.33 ^c^	−0.29 ^a^	0.001	0.13 ^a^	0.23 ^a^	−0.26 ^b^	<0.001
16	0.69 ^a^	−0.13 ^b^	0.04 ^b^	0.005	0.53	0.04	0.11	0.009
20	0.26	0.16	0.24	0.674	0.37	0.09	0.17	0.073
28	0.51	0.21	1.20	0.020	0.35	0.19	1.00	0.039
32	0.07	-	0.18	0.186	−0.06	-	0.21	0.001
36	0.00	-	0.00	0.710	0.11	-	−0.07	0.001
38	0.04	-	-		0.00	-	-	
**Second Vaccination Cycle**								
4	−0.24	-	−0.23	0.934	−0.13	-	−0.14	0.804
8	−0.22	-	−0.17	0.509	−0.17	-	−0.09	0.186

For each dimension, the different superscript letters in the same row represent significant differences between the means.

**Table 2:** Comparison of post-mortem morphometry between groups (mean ± SEM) (least squares model).

Item	Group C	Group E2	Group L2	Group L3	*p*-Value ^1^
	*n* = 11 (57w)	*n* = 8 (57w)	*n* = 6 (37w)	*n* = 11 (57w)	
*Carcass weight (kg)*	132.6 ± 4.7	123.1 ± 6.1	113.0 ± 4.4	117.2 ± 3.8	0.412
Aggregated weight (g)	829.7 ± 42.5	259.6 ± 45.9	375.7 ± 48.2	227.3 ± 38.3	0.103
Testis weight (g)	314.4 ± 17.1	85.6 ± 18.5	140.0 ± 19.5	77.0 ± 15.5	0.065
Epididymal weight (g)	10.5 ± 4.6	44.3 ± 4.9	42.6 ± 5.2	36.6 ± 4.1	0.741
Testis length (cm)	12.4 ± 0.4	8.0 ± 0.5	8.5 ± 0.5	7.8 ± 0.4	0.320
Testis width (cm)	7.7 ± 0.3	5.9 ± 0.3	4.7 ± 0.3	5.6 ± 0.2	0.132
Epididimal length (cm)	20.0 ± 0.7	14.2 ± 0.7	16.0 ± 0.7	13.7 ± 0.6	0.133

Aggregated weight represents the weight of the testicle and appended structures retrieved from the visceral layer of the vaginal tunica. ^1^ Comparison between the treated groups only.

## Data Availability

The data presented in this study are openly available in Mendeley Data at (doi:10.17632/399rpcc49r.2).
